# EMDR treatment for people with intellectual disabilities: a systematic review about difficulties and adaptations

**DOI:** 10.3389/fpsyt.2023.1328310

**Published:** 2024-01-11

**Authors:** Simone M. Schipper-Eindhoven, Nanda C. de Knegt, Liesbeth Mevissen, Jos van Loon, Ralph de Vries, Majlinda Zhuniq, Marrie H. J. Bekker

**Affiliations:** ^1^Zodiak, Prinsenstichting, Purmerend, Netherlands; ^2^Department of Clinical Psychology, Vrije Universiteit, Amsterdam, Netherlands; ^3^Psychotrauma Practice, Rha, Netherlands; ^4^Department of Special Needs Education, Ghent University, Ghent, Belgium; ^5^Vrije Universiteit, Medical Library, Amsterdam, Netherlands; ^6^Department of Clinical Psychology, Biological Psychology, and Psychotherapy, University of Mannheim, Mannheim, Germany

**Keywords:** eye movement desensitization and reprocessing, EMDR, post-traumatic stress disorder, PTSD, intellectual disabilities, ID, trauma

## Abstract

**Introduction:**

People with intellectual disabilities (ID) are at increased risk for developing Post Traumatic Stress Disorder (PTSD). Emerging evidence indicates that Eye Movement Desensitization and Reprocessing (EMDR) therapy is feasible and potentially effective for this group. However, communication, cognition, stress regulation, and attachment difficulties may interfere with the EMDR process. Adaptation of the EMDR protocol seems therefore required for this population.

**Aim:**

This review aims to systematically identify and categorize the difficulties in applying EMDR to people with ID and the adaptations made by therapists to overcome these challenges.

**Methods:**

A literature search was performed in May 2023. Article selection was based on inclusion and exclusion criteria and quality appraisal.

**Results:**

After screening, 13 articles remained for further review. The identified difficulties and adaptations were categorized into the three domains of adaptive functioning (i.e., conceptual, social, and practical functioning). Considerable difficulties in applying the EMDR protocol for this group were reported. The adaptations made by therapists to overcome these difficulties were highly variable. They could be divided into three main categories: adaptions in EMDR delivery (e.g., tuning to the developmental level of the client, simplifying language, decreasing pace), involvement of others (e.g., involving family or support staff during or in between sessions), and the therapeutic relationship (e.g., taking more time, supportive attitude).

**Discussion:**

The variability of the number of mentioned difficulties and adaptations per study seems to be partly related to the specific EMDR protocol that was used. In particular, when the Shapiro adult protocol was administered, relatively more detailed difficulties and adaptations were described than in publications based on derived existing versions of an EMDR protocol for children and adolescents. A probable explanation is that already embedded modifications in these protocols facilitate the needed attunement to the client’s level of functioning.

**Practical implications:**

The authors of this review suggest that EMDR protocols for children and adolescents could be adapted for people with an intellectual disability. Further research should focus on the involvement of trusted others in EMDR therapy for people with ID and the therapeutic relationship from an attachment and relational-based perspective.

## Introduction

Children and adults with intellectual disabilities (ID) are at increased risk for adverse life experiences and trauma-related mental health problems (1, 2). ID is defined in the Diagnostic and Statistical Manual of Mental Disorders (DSM-5) as a condition characterized by significant limitations in both intellectual and adaptive functioning (3). An IQ score of 70 or below means that there is a significant cognitive deficit. Adaptive functioning includes the conceptual, social, and practical domains (3). These domains contain skills that are learned and performed by people in their everyday lives and can be assessed with instruments such as Vineland-3 and the ADaptive Ability Performance Test (ADAPT) (3–5). The conceptual domain involves skills regarding memory, language, problem-solving, and judgment in novel situations. The social domain involves skills concerning awareness of others’ thoughts, feelings, and experiences; empathy, and communication. Finally, the practical domain contains skills concerning learning and self-management across life settings.

In the common population, the lifetime prevalence of post-traumatic stress disorder (PTSD) is estimated at 5–10% (6, 7). For individuals with ID, however, the prevalence rates of PTSD vary between 10 and 40% and are even higher in, for example (forensic) mental healthcare settings (8, 9). This relatively high risk for developing PTSD for people with ID appears to be caused by three factors.

First, people with ID are more frequently exposed to interpersonal traumatic events, such as physical, sexual, and emotional violence (10, 11). Second, a lower level of cognitive functioning is a well-known risk factor for the development of PTSD after traumatic exposure (12–14). Third, people with ID may also be more susceptible to the effects of upsetting and traumatic experiences due to impairments in stress regulation and adaptability (15, 16).

Despite the increased risk of developing PTSD, trauma-related symptoms often remain undiagnosed and untreated (17). This is partly caused by diagnostic overshadowing: classifying the core of the individual’s difficulties as a consequence of ID rather than trauma, leading to referral toward behavior-based rather than trauma-based therapies (18). Furthermore, limited cognitive and verbal abilities can hinder traditional treatment approaches that often rely on verbal communication (19, 20). Successful engagement in trauma-based therapy might also be complicated by challenges in establishing and maintaining therapeutic relationships with people with ID (21). The challenges are caused by an increased risk of attachment difficulties, such as interpersonal distrust, overly depending on other persons for support and preoccupation about possible abandonment (22).

The fact that PTSD is over-represented and poorly treated in the ID population requires feasible, safe, and effective trauma-focused psychological interventions for this specific group (23). For the treatment of PTSD in the common population, trauma-focused Cognitive behavioral therapy (CBT) and Eye Movement Desensitization and Reprocessing (EMDR) therapy are recommended by the World Health Organization (WHO, 2013) and the National Institute for Clinical Excellence (NICE, 2018) guidelines (24). EMDR therapy might be a more suitable intervention for people with ID and PTSS because it is less reliant on verbal abilities than CBT (25, 26). The focus of this review therefore will be on EMDR therapy.

EMDR therapy is a protocolled, psychotherapeutic approach developed by Shapiro (27). It aims to resolve symptoms related to disturbing and unprocessed life events. According to Shapiro’s Adaptive Information Processing Model (AIP) experiencing a traumatic event might coincide with insufficient information processing and a lack of integration with existing memory networks. This results in the memory of the traumatic event being stored in its “raw” form along with associated disturbed thoughts, sensations, and emotions. The traumatic memory remains unprocessed which results in symptoms of PTSD and a wide range of other disorders (26).

EMDR therapy consists of a structured eight-phase method. Phase 1 encompasses history-taking and case formulation. Phase 2 is the preparation phase; the client is prepared for the therapy. Phase 3 focuses on determining the target memory. During phases 4–6, memory processing to adaptive resolution takes place. An important part of the procedure is the performance of a working-memory-demanding task, for example, the therapist moves his/her fingers back and forth in front of the participant and asks him/her to track the movements, while the participant focuses on the trauma memory. Repeatedly, the participant is asked to report about emotional, cognitive, and somatic sensations that appear. Distress level during this traumatic memory recall is measured using subjective units of distress (SUD). The installation phase (5) aims to associate positive cognition with a traumatic memory to avoid distressing dysfunctional responses. The Validity of Cognition (VOC) scale is applied to measure whether the participant truly believes this positive cognition. Phase 7 is dedicated to closing down the session and preparing the participant for the period between sessions. Phase 8 consists of re-evaluation and integration (26). Although the EMDR protocol does not provide an explicit description of the required therapeutic relationship, Shapiro (26) mentions the importance of establishing a firm therapeutic alliance. The client and clinician should have established a sufficient level of trust before EMDR processing should be initiated, and the clinician should offer compassion and unconditional support.

The standard EMDR protocol can be adapted for children and adolescents, necessitating modifications in language, tools, and technical aspects to align with their cognitive and developmental abilities (28, 29). The eight phases of the EMDR protocol are applied with considerations for the child’s age, level of development, and life context. Emphasis is placed on assessing explicit and nonverbal communication. The phases involve establishing a therapeutic relationship, explaining EMDR, determining family involvement, target identification, desensitization, adaptive resolution, positive cognition installation, body scan, closure, and re-evaluation. For children, target selection and bilateral stimulation may involve special objects, toys, drawings, or other therapeutic tools (28, 29).

The effectiveness of EMDR in PTSD has been established in various meta-analyses (30–32). According to NICE guidelines, EMDR is indicated in the treatment of PTSD for adults and should also be considered for children and young people aged 7 to 17 years. Research suggests that EMDR therapy could also be effective for children and adolescents with PTSD (33). NICE guidelines, however, lack information concerning effective treatment for PTSD for people with ID. Emerging evidence indicates that EMDR is feasible and potentially effective for people with ID and PTSD (23, 34–37). Nonetheless, according to a randomized controlled feasibility trial conducted by Karatzias et al., EMDR, although generating improvements in PTSD symptoms, showed no significant improvement compared to standard therapy (38). Unfortunately, scientific evidence in this field is limited and fragmented, with only a few controlled case studies, including the one mentioned above by Karatzias and colleagues. Furthermore, these studies differ in their study design, population characteristics, and the use of the (adapted) EMDR protocol, making it challenging to assess their effectiveness (23). Four literature reviews on the effectiveness of EMDR for people with ID were conducted in the past years (15, 23, 34, 39). These studies suggest that EMDR may be an effective treatment for people with ID and PTSD. However, in their scoping review, Smith and colleagues suggest that EMDR in people with ID may be less efficacious than previously thought. Moreover, they notice, between studies, an apparent difference in the protocols used and adaptations in administering EMDR. Therefore, Smith and colleagues highlight the need for future research, to develop a reliable EMDR protocol suitable for this target group.

As stated before in this introduction, successful engagement in psychological (trauma) therapy for people with ID is complicated due to cognitive deficiencies, limited social and communicative skills, and attachment difficulties (22, 40, 41). We assume that these problems would also influence the process of EMDR therapy for people with ID and that adaptations are therefore required. Creating a safe and accepting therapeutic relationship, an important precondition for conducting EMDR (26) may form a particular challenge in EMDR therapy for people with ID. Their less effective coping strategies and attachment difficulties could demand more support in reducing stress during the recall of traumatic memories (22, 42). People with ID are often more likely to have relationships based on practical support than on emotionally focused support (43). Further, their position in relationships is weakened by difficulties in communication and comprehension. Developing a therapeutic relationship in which the client’s emotions are the focus, will therefore be challenging (43).

To our awareness, no systematic literature overview is available that specifically addresses these challenges regarding the delivery of EMDR to people with ID as described in our introduction (e.g., cognitive and communicative impairments, difficulties with attachment, and coping strategies), as well as possible solutions and adaptations. A detailed overview of difficulties related to, and adaptations regarding the use of the EMDR protocol for people with ID can be relevant to overcome challenges and thereby improve the acceptability and quality of EMDR treatment and therapy outcomes for this group (16). Additionally, such information might contribute to constructing a reliable EMDR protocol suitable for people with an ID that could be used in further research. This study aims, therefore, to systematically identify and categorize the difficulties in applying EMDR to people with ID and adaptations made so far by therapists to overcome these challenges.

## Methods

### Literature review

This systematic literature review is reported according to the Preferred Reporting Items for Systematic Reviews and Meta-Analyses (PRISMA) (44).

### Search strategy

To identify all relevant publications, systematic searches were conducted in the bibliographic databases PubMed, Embase, APA PsycInfo (Ebsco), and Web of Science (Core Collection) from inception up to May 1, 2023, in collaboration with a medical information specialist. The following terms were used (including synonyms and closely related words) as index terms or free-text words: “Eye Movement Desensitization,” “Intellectual Disability,” and “Cognitive Dysfunction.” Duplicate articles were excluded using Endnote X20.0.1 (Clarivate^™^), following the Amsterdam Efficient Deduplication (AED)-method (45) and the Bramer method (46). The full search strategies for all databases can be found in the Supplementary material.

### Selection process

Three authors (SS, NdK, LM) independently screened all potentially relevant titles and abstracts for eligibility. Differences in judgment were resolved through discussion until a consensus was reached. Studies were included if they met the criteria in Table 1. The full text of the selected articles was obtained for further review. The same three authors did an additional full-text screening. Differences in judgment were, again resolved by discussing them until consensus was reached. Studies were only included if they met the criteria displayed in Table 2.

**Table 1:** Inclusion and exclusion criteria for EMDR treatment for people with intellectual disabilities.

Inclusion criteria	Exclusion criteria
Articles published in peer-reviewed journals; such as cross-sectional, case–control, case series, and process evaluation studies	Published commentaries and letters, literature reviews, conference proceedings, chapters, and dissertations
The publication year 1989 (first publication on EMDR by Shapiro) – 2022	Publication year older than 1989
Written in the English language	Written in other than the English language
Human studies	Animal studies
Intellectual disability (synonyms and related words were included)	Developmental disabilities without intellectual disabilities, learning disabilities, mental handicaps when mentioned without intellectual disabilities, psychiatric disorders without intellectual disabilities, Alzheimer’s/dementia, cognitive defect
Eye movement desensitization and reprocessing (synonyms that were included: EMDR)	Articles that did not include any of the two terms used or EMDR was used in intensive clinical treatment combined with CBT and exposure

EMDR, Eye Movement Desensitization Reprocessing; ID, Intellectual Disabilities.

**Table 2:** Inclusion and exclusion criteria full text for EMDR treatment for people with intellectual disabilities.

Inclusion criteria	Exclusion criteria
Addresses applicability EMDR with ID target group	No comments about the applicability of EMDR with ID target group in the article
At least one difficulty/adaptation applying EMDR with the ID target group is described	No difficulty or adaptation applying EMDR with the ID target group is described

EMDR, Eye Movement Desensitization Reprocessing; ID, Intellectual Disabilities.

The three authors then independently evaluated the methodological quality of the full texts using the JBI Critical Appraisal Checklist for Case Series (47). This checklist includes 10 questions addressing the internal validity, risk of bias in case series designs (e.g., confounding, selection, and information bias), and report clarity. The following quality thresholds were used: low quality (0–33% of criteria met), medium quality (34–66% of criteria met), and high quality (at least 67% or more of criteria met). Table 3 presents details of the quality assessment. A second quality appraisal was performed using the Mixed Methods Appraisal Tool, MMAT 2018 (53). This tool addresses the methodological quality of five categories of studies: qualitative research, randomized controlled trials, non-randomized studies, quantitative descriptive studies, and mixed methods studies. Only studies of medium and high quality were included for further analyses. Differences in judgment were resolved through consensus.

**Table 3:** Overview of reviewed articles on EMDR for people with ID: findings and quality appraisal.

Authors/Date	Type	EMDR protocol	Quality appraisal JBI	Quality appraisal MMAT
Barrol and Seubert, 2010	Multiple case study	Adult EMDR protocol (27)	6 /10 Medium quality	100%^a^
Barrowcliff and Evans, 2015	Single case study	Adult EMDR protocol (27)	5/10 Medium quality	80%
Dilly, 2014	Single case study	Adult EMDR protocol (27)	5/10 Medium quality	100%
Karatzias et al., 2019	Randomized feasibility trial	Adult EMDR protocol (27)	NA	80%
Mevissen et al., 2011 (1)	Multiple case study	Adapted Dutch translation of Shapiro’s protocol (48)	7/10 High quality	100%
Mevissen et al., 2011 (2)	Multiple case study	Adapted Dutch translation of Shapiro’s protocol (48)	5/10 Medium quality	60%
Mevissen et al., 2012	Multiple case study	Storytelling (49)	6/10 Medium quality	100%
Mevissen et al., 2017	Multiple case study	Dutch protocol for children and adolescents (50)	7/10 High quality	100%
Pennix Quevedo et al., 2021	Multiple case study	Dutch protocol for children and adolescents (50)	8/10 High quality	100%
Porter, 2021	Multiple case study	Adult EMDR protocol (27)	1/10 Low quality	20%
Rodenburg et al., 2019	Single case study	Children’s EMDR protocol (51)	6/10 Medium quality	100%
Unwin et al., 2019	Acceptability study	Adult EMDR Protocol (26)	NA	80%
Unwin et al., 2023	Feasibility study	2 phase model: Psychoeducation and stabilization (PES) phase EMDR protocol based on a combination of adult (27), child (49) and attachment-focused EMDR (52)	NA	60%
Verhagen et al., 2022	Multiple case study	Dutch protocol for children and adolescents (50)	9/10 High quality	100%

EMDR, Eye Movement Desensitization Reprocessing; ID, Intellectual Disabilities; JBI, JBI Critical Appraisal-Checklist for Case Series; MMAT, Mixed Methods Appraisal Tool 2018; NA, Not Applicable. ^a^% of MMAT quality criteria met.

### Data extraction and assessment

The first author screened each included study for explicitly mentioned difficulties and adaptations in applying EMDR to people with ID. Subsequently, findings over all studies were combined, labeled according to the corresponding phase in the EMDR process, and categorized into the three domains of adaptive functioning: the cognitive/conceptual, social, and practical domains (3).

## Results

### Search results

**Figure 1:**
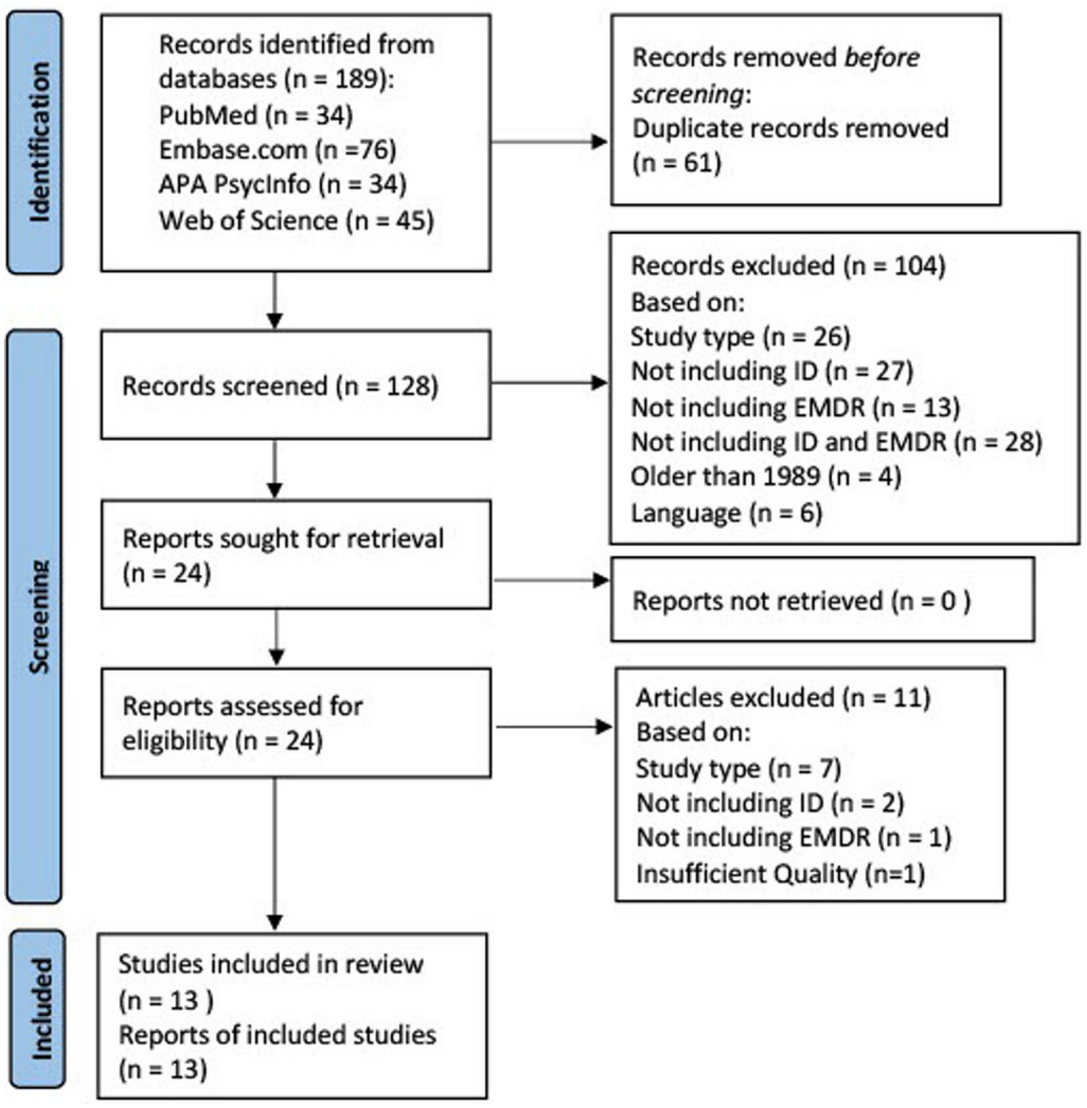
Flowchart of the search and selection procedure of articles.

The literature search generated a total of 189 references. After removing duplicates 13 references remained. Figure 1 displays the flowchart of the search and selection procedure.

In total, 13 articles were analyzed after exclusion: 10 case studies, one randomized feasibility trial, one acceptability, and one feasibility study. Tables 3–7 summarize our findings. Table 3 gives an overview of the included study, the study characteristics, the EMDR protocol that was used, and the results of the quality appraisal. The results of the quality appraisal show that four of the case studies yielded a methodological high quality on JBI and a 100% MMAT score. Another four case studies were considered to have a medium quality on JBI and a 100% MMAT score. The two remaining case studies reached a JBI medium quality and an MMAT score of, respectively, 80 and 60%. The RCT (29 participants, effect size ηp^2^ = 0.07–0.22) obtained a 100% MMAT score. The acceptability and feasibility studies were granted an MMAT score of, respectively, 80 and 60%. After the quality appraisal, one study of low quality, Porter (2022), was excluded. A detailed overview of the quality appraisal can be obtained through the first author of this review.

In the included studies, EMDR was administered by therapists with various levels of training and experience, both in EMDR itself and in working with individuals with ID. The provided EMDR therapy was based on the Shapiro EMDR Adult or Child protocols (26), the Dutch translation of Shapiro’s protocol (48), or the Dutch protocol for children and adolescents (50), including the Storytelling method (49). In addition, we found high variability in the number of EMDR sessions offered (between 3 to 25 sessions) and the length of the sessions (45 up to 120 min).

Most studies found positive effects of EMDR. Table 4 displays a detailed overview of the difficulties in administering EMDR to people with ID described in each study and the corresponding adaptations that therapists made to overcome these difficulties. The numbers of mentioned difficulties and adaptations vary between studies and seem to be partly related to the specific EMDR protocols that were used. Notably, in papers referring to EMDR with the Shapiro adult protocol (2001) (16, 18, 38, 54, 55), relatively more detailed difficulties and adaptations were described than in publications referring to EMDR with a version of the child’s EMDR protocol (35–37, 56–59). The publication by Unwin et al. introduces a significant modification to the EMDR protocol; the addition of a phase called psycho-education and emotional stabilization (PES) (25).

**Table 4:** Identified difficulties and corresponding adaptations in EMDR for people with ID per included Study.

Authors/Date	Difficulty in EMDR for people with ID per included Study	Corresponding adaptation
Barrol and Seubert, 2010	C is preoccupied with more recent traumatic events instead of traumatic memories from childhood	Target selection is based on the most pressing event for C
	C is not able to process past events and connect them to present triggers	Target selection is based on hypotheses of T
	C has difficulty tracking eye movements	An electronic tactile-auditory system or tapping is used
	There is a returning disturbance on target in the next session, after SUD = 0 in prior session	Treatment on the same target is repeated
	C has short attention span and inability to multitask	More verbal encouragement from T is needed T repeats directives
	Lack of communication skills of C	T gives more suggestive and active interventions More time is reserved for gathering history from C and the support system The language for negative and positive cognitions is adapted
	Processing phase is too overwhelming for C	Staff is present during a session using a tapping form of BLS Resources are used more frequently The painful event is titrated by breaking the memory into smaller, more manageable parts C is more frequently directed from dysfunctional material to positive resources
	C is not able to report updates	A trusted other provides support during sessions
	C is resistant to work on trauma related memories	More time is invested in building therapeutic rapport
	C is dependent on support from others	Family and direct care staff receive psychoeducation
	C has cognitive deficits	The SUD and VoC scales are written out or communicated by means of physical gestures in order to make them more concrete
	C is unaware of relationship between emotions and events	Emotional and somatic material is being processed without a narrative content
	C has problems in generalizing learning, sustaining new learning	The Recent Events Protocol (27) is used
Barrowcliff and Evans, 2015	C has poor communication skills client and main caregiver is traumatized	A family member of the main caregiver provides historical details
	C is rapidly overwhelmed	The distancing technique is used
	The installation of safe place requires more time/practice	Concrete exemplars, such as an object, are utilized
	C lacks the ability to verbalize events according to cognitions and emotions	T uses supported labeling
	C is unable to complete SUD and VoC ratings and unable to indicate “more” or “less” by typical methods of adaptation	C reports by using “better” or “worse”
	C has a visual impairment	Alternating hand-taps are used as BLS
	C has difficulties in engaging in lateral stimulation while providing verbal feedback of changes and emotional responses	T observes discernible levels of distress of C
	C finds the use of a number scale too difficult	T uses verbal cues of “better” or “worse” along with observation
	The difference in scales between the SUD and the VoC causes confusion for C	T uses congruent scales
	C has limited expressive language	T uses visual formats
Dilly, 2014	C’s level of arousal during sessions is too high	Focus is on safe place visualization techniques
	C needs to receive additional support	T informs members of the clinical team about sessions
	C has a lack of communication skills	T uses symbol cards, pictures, and drawings
Karatzias et al., 2019	C needs to feel safe/connected	One therapist is involved throughout the treatment
	C has a lack of communication skills	T uses adjusted patient information, such as symbolized information, easy-read versions, or a short video demonstrating the delivery of EMDR
	C has problems to attend therapy appointments	Families and support workers are initiated in the recruitment process at an early stage
	C experiences too much distress	A family member or supporter is present during therapy
	C is more dependent on support from others in daily life	T provides information for participant and family/support staff to help cope with recurring memories related to the trauma between sessions
	C has a physical impairment such as cerebral palsy and visual and hearing problems	Individual adjustments of type of BLS are made
Mevissen et al., 2011 (1)	During history taking no painful memories were identified at first	T performs a further detailed analysis of trauma timeline with parents
	C experiences lack of: •Sense of safety •Communication skills •Integration of therapeutic process in daily life	C is accompanied by a parent or professional caregiver during treatment
	The standard EMDR protocol is not suitable	The EMDR protocol is adapted to the level of clients’ cognitive and emotional functioning Parts of the protocol that appear to be too difficult are eliminated
	C has cognitive disabilities and poor verbal capacities	T’s attitude is more directive without taking over T simplifies language
	C is unable to perform eye movements	Other forms of BLS are used, primarily bilateral sound and tactile stimulation
	C needs to relax and feel safe	Between the story narrations, an enjoyable game is played to emphasize safety and empowerment in the present time
Mevissen et al., 2011 (2)	C has cognitive disabilities and poor verbal capacities	First session is used to initiate preparation for treatment with a trusted family member and caregiver Complaint-related changes are reported by the caregiver at the beginning of each session
	NA	A family member is present for each EMDR session
Mevissen et al., 2012	C is neither able to speak, nor create drawings	The Story Telling Method is employed
	C could not tolerate headphones, buzzers, or eye movements	T uses external audio speakers
	Parents of C are traumatized themselves which complicates their participation in the EMDR process	Parents are treated with EMDR before the treatment of C
Mevissen et al., 2017	NA	Visual timeline of adapted ADIS-C PTSD section supports decision about the sequence of targeting memories
	C does not understand instructions	T omits rating of VoC
	C has cognitive disabilities and poor verbal capacities	Mental age-related instructions of the Dutch EMDR protocol for children and adolescents without intellectual disability are used with visual analog scales for SUD and VoC
	C shows avoidance behaviors	T has a directive and supportive attitude
Pennix Quevedo et al., 2021	NA	The EMDR eight-phase protocol for children and adolescents is used with omission of installing a safe place or any other form of stabilization
	Presence of important traumatic memories are missed by T or C avoids reporting them	Authors suggest that not the disturbance related to the target images, but a decrease in PTSD symptomatology should be used as a criterion for completing the treatment
Rodenburg et al., 2019	C has cognitive disabilities	Children’s protocol is used to facilitate the use of drawings and storytelling
	C has poor verbal capacities	Parents participate in the treatment to verbalize C’s emotions and cognitions
Unwin et al., 2019	C has current issues to work on	T reserves time to talk about present issues during session
	C is more susceptible to the views of family members who may also be worried about bringing up the past and/or trying out a new kind of therapy	T involves family
	C has difficulties in understanding the complex mechanisms involved in EMDR and lack the impetus for reliving	NA
	C has a fear of destabilizing	T spends more time to build resources, develop self-management techniques, and improve emotional awareness and resilience
	The reduced talking in EMDR makes it more difficult to develop an empathetic therapeutic relationship	T uses EMDR in combination with other more verbal approaches
	T does not feel equipped to adapt the protocol to suit C	NA
	Participants experience elements of EMDR to be difficult, and intrusive and this affects engagement with therapy	T adapts protocol to individual needs T has a directive and prompting attitude Distress and Positive Cognition Scales are simplified
Unwin et al., 2023	BLS is unfamiliar for C and its purpose hard to explain	Psychoeducation and Stabilization (PES) phase prior to EMDR
	C has complicated trauma history	PES phase prior to EMDR
	Goal setting difficult due to no clear and defined trauma memories	T uses EMDR techniques developed for children
	C goals are often not trauma focused which leads to reluctance to address trauma	PES phase prior to EMDR
	C has a fear of feeling overwhelmed	PES phase prior to EMDR
	C has the need to address other issues	T reserves time to talk about present issues, thus needing more sessions
	For the remote delivery of EMDR, C experiences problems with access to and confidence with IT facilities	C needs support of staff and IT department organization
	For the remote delivery of EMDR C is in need for a safe space to work from and support if distressed	C receives support of staff
Verhagen, 2022	C has limited cognitive and emotional abilities	Dutch EMDR protocol for children and adolescents up to 18 years (50) is used

EMDR, Eye Movement Desensitization Reprocessing; ID, Intellectual Disabilities; C, Client with ID; T, EMDR therapist; SUD, Subjective Unit of Disturbance; BLS, Bilateral Stimulation; NA, Not Applicable; VoC, Validity of Cognition; ADIS-C, Anxiety Disorder Interview Schedule-Child; PTSD, Post-Traumatic Stress Disorder, PES, Psychoeducation and Stabilization.

In the following analyses, the first author (SS) categorized the difficulties and adaptations described in Table 4 into the three domains of adaptive functioning (3): the cognitive/conceptual, social, and practical domains (Tables 5–7, respectively). Subsequently, the difficulties and adaptations were classified by the first and third author (SS and LM) into the corresponding phase in the EMDR process (26). In the cognitive/conceptual domain (Table 5), the identified difficulties that may hinder EMDR therapy for people with ID include a lack of verbal and communication skills; a short attention span; an inability to multitask; a lack of abstract thinking, and difficulties in making connections between past and current behaviors. To overcome these difficulties, the parts of the protocol that appear to be too difficult for these reasons, are eliminated (18, 36, 57). In addition, a protocol for EMDR with children and adolescents is often used, which is adapted to the client’s developmental age level (35–37, 50, 59). This includes the ‘storytelling method’ developed by Lovett for applying EMDR to children under 3, where parents/caregivers narrate the traumatic event (58). Other adaptations in this domain include the use of simplified language and materials, and/or focusing on emotional material and physical sensations without cognitive/narrative content (18, 38, 54, 55). Furthermore, the EMDR therapist adapts his/her behavior by displaying a more directive attitude and greater verbal involvement (18, 54, 57). All the articles identified in this review highlight the significant role played by the support system, which includes family members and caregivers. They participate in the therapy sessions in various ways, such as providing information, verbalizing the person’s emotions and thoughts during the sessions, or acting as co-therapists to support communication. This adaptation seems crucial in this domain and is present in all the articles reviewed. All aforementioned adaptations in the cognitive/conceptual domain are applied during all phases of the EMDR protocol.

**Table 5:** Categorization of identified difficulties and corresponding adaptations in EMDR for people with ID relating to the cognitive and conceptual domain.^a^

Difficulties relating to the cognitive/conceptual domain	Corresponding adaptations relating to the cognitive/conceptual domain	Corresponding phase EMDR protocol^b^
Lack of verbal and communication skills of C: C is not able to report updates C lacks the ability to verbalize events according to cognitions and emotions C is not able to identify trauma related memories The presence of important traumatic memories may be missed by T C is less able to provide a detailed history of the trauma and early life events C has difficulty with verbally describing targets	Participation of family and/or caregivers during the whole EMDR process: To gather information about history or complaint-related changes and timelines	1
	To communicate the rationale for use of EMDR	1
	As a co-therapist during sessions to support communication and observation	1–8
	To verbalize the person’s emotions and cognitions during sessions	1–8
	Adaptation EMDR protocol to the level of the client’s developmental/mental age:	
	Use of a protocol for children/adolescents including the storytelling method	1–8
	Eliminate parts of the protocol that appear to be too difficult	1–8
	Adding Psychoeducation and Stabilization (PES) module before EMDR that focuses on establishing strengths and resources, stabilizing emotional regulation, and founding alliance and trust	1–3
C has a short attention span Ca has an inability to multitask	T displays a more directive, suggestive, and active therapeutic attitude with greater verbal involvement and supportive labeling	1–8
C has a lack of abstract thinking: C is unable to complete SUD and VoC ratings C is unable to indicate “more” or “less” by typical methods of adaptation C does not understand the need to address past C has difficulties with imaginal techniques	T simplifies language by: Adaption of language of the negative and positive cognitions congruent with the developmental age of the client The use of simplified SUD and VoC scales	1–8
	T uses visual communication aids, drawing, and gestures	1–8
	Observation of discernible levels of distress of C by T	3–6
	Not the disturbance related to the target images but a decrease in PTSD symptomatology should be used as a criterion for completing the treatment	8
	T spends more sessions on gathering a clinical history, teaching resources, and supporting clients to understand the EMDR process	1
C has difficulties making connections between past/history and current behaviors/emotions: C is preoccupied with recent events instead of past traumatic memories C is not able to process past events or connect them to present triggers C is unaware of the connection between emotions and events	T focuses on the traumatic event that is immediately relevant to the client (as opposed to actively seeking worst or touchstone events)	3
	T uses of the Bridge Back technique	3
	T focuses on processing emotional material and physical sensations without cognitive/narrative content	3–4

EMDR, Eye Movement Desensitization Reprocessing; ID, Intellectual Disabilities; C, Client with ID; T, EMDR therapist; PES, Psychoeducation and Stabilization; SUD, Subjective Unit of Disturbance; VoC, Validity of Cognition; PTSD, Post Traumatic Stress Disorder. ^a^The cognitive and conceptual domain is the first domain of adaptive functioning (3), ^b^Corresponding phase of EMDR protocol (26).

**Table 6:** Categorization of identified difficulties and corresponding adaptations in EMDR for people with ID relating to the social domain^a^.

Difficulties relating to the social domain	Corresponding adaptations relating to the social domain	Corresponding phase EMDR protocol^b^
C experiences a lack of sense of safety: C shows avoidance behaviors C has difficulty with social contact C is resistant to work on trauma related memories T has problems developing a therapeutic relationship with C	Having a trusted other, like a (parent or caregiver) present during sessions	1–8
	One continuous clinician is involved throughout the treatment	1–8
	T spends more time on building therapeutic rapport	1–8
	T shows greater verbal involvement during the trauma processing	3–7
	T uses games in between trauma processing to make C feel more relaxed and to emphasize safety and empowerment	4
	T focuses on safe place visualization techniques	2–7
	Adding Psychoeducation and Stabilization (PES) module before EMDR that focuses on establishing strengths and resources, stabilizing emotional regulation, and founding alliance and trust	1–3
C is more susceptible to the views of important others: Important others are worried about bringing up the past Important others are traumatized themselves Important others are worried trying out a new kind of therapy	T offers EMDR for parents or staff to enable them to participate in treatment	1–2
	Information within sessions is shared with members of the clinical team to receive additional support	BS
	T provides information materials to help cope with recurring memories related to the trauma under treatment, and provides details on local support between therapy sessions	1
C has a lack of stress management skills: C is rapidly overwhelmed C shows a high level of arousal during sessions C has a fear of destabilizing C has poor affect awareness C is getting too agitated The processing phase too intense for C	T spends more time to build resources to recognize strengths, develop self-management techniques, and improve emotional awareness and resilience	1–2
	T adds PES module	1–3
	T checks SUD more frequent	4
	T directs C more frequently from dysfunctional material to positive resources	2–7
	T titrates the painful event by breaking the memory into smaller, more manageable parts	3–4
	A trusted other is present during the sessions using a tapping form of BLS	4
	Tapping BLS is used outside sessions to help to calm C down	BS
	T uses the distancing technique	3–4
Often complicated trauma history	T adds PES module	1–3
C has a lot of current issues to work on	T reserves time for present issues	3

EMDR, Eye Movement Desensitization Reprocessing; ID, Intellectual Disabilities; C, Client with ID; T, EMDR therapist; PES, Psychoeducation and Stabilization; BS, Between Sessions; SUD, Subjective Unit of Disturbance; BLS, Bilateral Stimulation. ^a^The social domain is the second domain of adaptive functioning (3), ^b^Corresponding phase of EMDR protocol (26).

**Table 7:** Categorization of identified difficulties and corresponding adaptations in EMDR for people with ID relating to the practical domain^a^.

Difficulties relating to the practical domain	Corresponding adaptations relating to the practical domain	Corresponding phase EMDR^b^ protocol
C experiences physical difficulties: C has problems with tracking eye movements C has a visual impairment C cannot tolerate headphones or buzzers	T uses adapted forms of BLS, such as: Bilateral sound Tactile stimulation Tactile-auditory electronic device	4
	Alternating hand-taps	
	External audio speakers	
In case of remote delivery of EMDR; C experiences problems with access to and confidence with IT facilities	C receives support of staff and the IT department of the organization	NA
C has an interdependency with caregiving systems: C has need for additional support C has problems to attend therapy appointments	T spends more focus and time on involvement, education, and support of family and direct care staff	NA
C experiences problems in generalizing learning: C has problems sustaining new learning C has problems integrating of the therapeutic process in daily life C shows a returning disturbance on target in next session after SUD = 0	T takes more time during all phases of therapy to make smaller steps or practice more: T repeats SUD check on past targets T repeats treatment on the same target T reserves more time for imagery practice of a safe space	1–8
	T checks the outcome of the memory processing with the “recent events protocol”(Shapiro, 2001)	8
	A parent or professional caregiver is present during treatment	1–8
	T provides adapted accessible information about PTSD and EMDR	1–2

EMDR, Eye Movement Desensitization Reprocessing; ID, Intellectual Disabilities; C, Client with ID; T, EMDR therapist; BLS, Bilateral Stimulation; IT, Information Technology; NA, Not Applicable; SUD, Subjective Unit of Disturbance; ^a^Practical domain is the third domain of adaptive functioning (3), ^b^Corresponding phase of EMDR protocol (26).

The social domain (Table 6) includes patient difficulties with feeling safe and/or developing social contact, which hinders the formation of a therapeutic relationship needed for engaging in EMDR. Regarding this domain, some authors (16, 18, 25, 38, 54, 55) also mention a lack of stress management skills, causing the person with ID to feel agitated or overwhelmed during the EMDR process. Furthermore, high susceptibility to the views of others and complex trauma histories are put forward as difficulties (16, 25). Many adaptations to cope with the difficulties in this domain also are identified, for all phases of the EMDR protocol. Examples are: having the accompany of a trusted other to give support during, and in between sessions; and taking more time to build the therapeutic relationship. Also, therapists employ greater verbal involvement, focus on positive resources, and teach affect management and self-soothing skills. In addition, EMDR was sometimes administered to parents who were traumatized themselves, enabling them to participate in treatment (58). The publication by Unwin et al. introduces a PES phase, which aims to build a strong alliance and trust between the client and therapist, stabilize emotional regulation, and install strengths and resources in the client (25).

Regarding the practical domain (Table 7), EMDR therapy for people with ID can be challenging due to physical impairments like visual or hearing deficiencies, or muscular problems. However, these challenges are overcome by using alternative forms of bilateral stimulation like tactile stimuli such as hand taps or buzzers or auditory ones such as sounds and headphones that suit the specific needs of the person (18, 38, 54, 57, 58). Another difficulty faced by individuals with ID in the practical domain is their struggle with generalizing learned skills from EMDR therapy to daily life. This is resolved by taking more time during all phases of EMDR therapy to enable smaller steps or to practice more (18, 57). Another challenge in the practical domain is the interdependence of individuals with ID and their caregiving systems. For instance, they may require extra assistance between EMDR therapy sessions or be unable to attend therapy appointments unaccompanied. Therefore, it is crucial to involve, educate, and support family members and direct care staff throughout all phases of EMDR therapy (16, 18, 25, 38, 55).

In conclusion, our findings revealed a wide range of adaptations that correspond to the three domains of adaptive functioning (3). These adaptations, which are presented in Tables 5–7, can be broadly categorized into three main groups: adaptations in EMDR delivery, adaptations in the engagement of others, and adaptations in the therapeutic relationship. Adaptations in EMDR delivery involve modifying the protocol to suit clients’ developmental levels, adjusting materials, adding or omitting certain elements, simplifying language, and adapting the pace of the sessions. Similarly, adaptations in the engagement of others include involving family members or support staff to facilitate communication and provide support during and between sessions or as a source of information. Finally, adaptations in the therapeutic relationship comprise elements such as taking more time to establish therapeutic rapport, increased verbal interaction, or a more supportive attitude from the therapist.

## Discussion

The current review aimed to systematically identify and categorize the difficulties described in the literature in applying EMDR to people with ID as well as adaptations made to overcome these challenges. Though findings suggest the potential effectiveness of EMDR with individuals with ID and PTSD, (23, 60) considerable difficulties in applying the EMDR protocol for this group are reported, corresponding to the three domains of limited adaptive functioning (3). The adaptations made by therapists to overcome these difficulties are highly variable and can be divided into three main categories: adaptations in EMDR delivery, in the engagement of others, and adaptations in the therapeutic relationship.

It appears that the number of difficulties and adaptations mentioned per study may be partly linked to the specific EMDR protocol utilized. In particular, when the Shapiro adult protocol (27) was administered, relatively more detailed difficulties and adaptations were described than in publications based on a version of the EMDR protocol for children (50). A probable explanation is that modifications are already embedded in these child protocols tailoring to the clients’ level of functioning, which for people with ID will not reach above a developmental level of 12 years. It is also possible that the high variability in difficulties and adaptations found between studies can be attributed to the diverse and variable nature of the group of people with ID. This variation implies that individuals with ID will have a broad range of abilities, needs, and challenges, which depend on the severity of their disability, social and communication skills, and level of attachment difficulties (22). Given this wide variability, EMDR therapy needs to be flexible and adapted to the individual’s unique characteristics and circumstances. Additionally, people with ID often experience interpersonal and/or complex trauma (10, 11), which may require modifications or additions to the EMDR protocol. For instance, it can be necessary to spend more time building therapeutic rapport and resources or to include a complete PES phase (18, 25). This concept of an extended EMDR treatment model is also demonstrated in the intensive inpatient trauma treatment program for families; KINGS ID (61).

The findings of our research align with the adaptations for Cognitive Behavioral Therapy (CBT) for individuals with ID as described by Dagnan et al. (62). The authors emphasize the importance of customizing CBT techniques to suit the specific needs of this group. This includes using activities instead of verbal interactions, simplifying processes, involving caregivers, adapting to the developmental level of the individual with ID, modifying language, being flexible, and using directive methods. The authors also highlight the significance of establishing a strong therapeutic alliance through rapport-building and communication strategies that are tailored to the individual’s cognitive abilities (62).

### Limitations

Regardless of our efforts to conduct this review systematically and rigorously, some limitations should be considered when interpreting the results. First, our focus had been on qualitative analysis and categorization of difficulties and adaptations of EMDR for people with ID, hindering a more quantitative comparison with findings from other studies such as Dagnan et al. (62). Second, the high variability of the design and population characteristics of the reviewed studies interferes with the generalizability of results. Depending on the severity of ID or the type of experienced trauma, some of the identified adaptations may be more applicable. On the other hand, this variability could also be considered a strength since this has led to a high number of identified difficulties and adaptations. Third, information was not constantly reported across the studies. Some studies did not provide detailed information regarding the level of training and experience of therapists using EMDR with individuals with ID, which may have influenced the reported difficulties. Additionally, some studies did not describe the EMDR protocol used in detail, making it harder to identify any adaptations. Moreover, many articles did not specify the exact number or duration of EMDR sessions, making it more challenging to identify possible adaptations in this area.

### Implications

From a clinical perspective, an EMDR child protocol [e.g., the EMDR protocol for children and adolescents (50)] seems to provide the best base for EMDR treatment of people with ID, because it encompasses a considerable amount of the identified adaptations and it is adjusted to these clients level of functioning. It is crucial that therapists have sufficient training and experience in both administering the EMDR protocol and in working with individuals with ID. Concerning EMDR delivery, the studies included in this review provide detailed descriptions of adaptations of the EMDR protocol. It is advisable to conduct further research to determine the most suitable adaptations, taking into account various subgroups defined by factors such as the severity of ID, physical limitations, and verbal capabilities (23). Focusing on the second category, the engagement of others, all studies described family or support staff as (more or less) active participants in the treatment. Involving family and/or support staff in the therapeutic process to provide comfort, support communication, and promote generalization of skills, seems a fundamental condition for an effective enrolment of individuals with ID in (EMDR) therapy (17, 62). The specific role of the family member or support staff, however, has not been explicitly defined in all studies. It seems important to clearly communicate the role of trusted others in EMDR sessions since their involvement can greatly influence the process. Also, there are often practical challenges in the continuity of carers, which makes it difficult for the same trusted other to be consistently present throughout therapy (21, 61). Finally, insight gained by the family member or support staff involved in the treatment could be an alternative explanation for the improvements reported (39). It is advisable to explore how trusted individuals can best contribute to each phase of the EMDR process and the amount of input they provide. Subsequently, the third category, the adaptations in the therapeutic relationship, might form the biggest challenge. It appears that there is currently a lack of explicit description of the therapeutic relationship required for EMDR therapy in research (63). According to Hase and Brisch, the therapeutic alliance should be described as an essential part of EMDR therapy. Since attachment theory offers a view on the development of interpersonal relationships in general, from their perspective, an attachment-based perspective of the therapeutic relationship required for EMDR would be desirable (63). This would be of even more importance for people with ID, especially those who struggle with attachment difficulties or complex or interpersonal trauma (21, 22, 64). A clear description of the necessary components of the therapeutic relationship in terms of speech, rhythm, eye contact, touch, and attunement of the therapist to the individual, could be a useful addition to the EMDR protocol for this specific group. Gentle Teaching (65), an attachment and relationship-based approach that focuses on building safe and loving alliances with people with ID, might be promising in pursuit of this objective. It could be worthwhile to incorporate elements of Gentle Teaching in the EMDR protocol to increase the accessibility and effectiveness of EMDR therapy for this group.

## Conclusion

The findings of this review show that, although research underlines the potential effectiveness of EMDR for individuals with ID and PTSD, applying the EMDR protocol to this population presents therapists with considerable difficulties in all three domains of these clients’ adaptive functioning. Adaptations in EMDR delivery, in the engagement of others, and in the therapeutic relationship are often required. Difficulties in EMDR delivery could largely be addressed by adaptations that are incorporated into EMDR protocols for children and adolescents. It is recommended to focus further research on how to involve trusted others in EMDR therapy for people with ID and on how to establish a therapeutic relationship from an attachment and relational-based perspective.

## Author contributions

SS-E: Conceptualization, Data curation, Methodology, Writing – original draft, Writing – review & editing. NK: Conceptualization, Data curation, Methodology, Writing – review & editing. LM: Conceptualization, Data curation, Methodology, Writing – review & editing. JL: Conceptualization, Methodology, Writing – review & editing. RV: Conceptualization, Methodology, Writing – review & editing. MZ: Conceptualization, Writing – review & editing. MB: Conceptualization, Methodology, Supervision, Writing – review & editing.
