# Supplementary material for: EMDR treatment for people with intellectual disabilities: a systematic review about difficulties and adaptations
Source: Front Psychiatry. 2024 Jan 11;14:1328310. doi: 10.3389/fpsyt.2023.1328310 (PMC10808451; doi:10.3389/fpsyt.2023.1328310)
Supplement: Supplementary file 1 [file Table_1.DOCX]

**Supplementary material EMDR Treatment for People with Intellectual Disabilities:**

**Full search strategy**

**PubMed Session Results (01 May 2023)**

| Search | Query | Items found |
| --- | --- | --- |
| #3 | **#1 AND #2** | 34 |
| #2 | **"Intellectual Disability"[Mesh] OR "Cognitive Dysfunction"[Mesh] OR "cognitive dysfunct*"[tiab] OR "intellectual disab*"[tiab] OR "intellectually disab*"[tiab] OR "mental disab*"[tiab] OR "mentally disab*"[tiab] OR "mental deficien*"[tiab] OR "mentally deficien*"[tiab] OR "intellectual deficien*"[tiab] OR "mental handicap*"[tiab] OR "mentally handicap*"[tiab] OR "Intellectual Development Disorder*"[tiab] OR "intellectual impair*"[tiab] OR "intellectually impair*"[tiab] OR "intellectual dysfunct*"[tiab] OR "mental impair*"[tiab] OR "mentally impair*"[tiab] OR "mental dysfunct*"[tiab] OR "cognitive impair*"[tiab] OR "cognitive disab*"[tiab] OR "cognitive deficien*"[tiab] OR "cognitive defect*"[tiab] OR "cognition impair*"[tiab] OR "cognition deficien*"[tiab] OR "Down Syndrome"[tiab] OR "Downs Syndrome"[tiab] OR "Down's Syndrome"[tiab]** | 242,306 |
| #1 | **"Desensitization, Psychologic"[Mesh:NoExp] OR "Eye Movement Desensitization Reprocessing"[Mesh] OR "Eye Movement Desensit*"[tiab] OR EMDR[tiab]** | 2,426 |

**Embase.com Session Results (01 May 2023)**

| Search | Query | Items found |
| --- | --- | --- |
| #3 | **#1 AND #2** | 76 |
| #2 | **'intellectual impairment'/exp OR 'cognitive defect'/exp OR 'cognitive dysfunct*':ab,ti,kw OR 'intellectual disab*':ab,ti,kw OR 'intellectually disab*':ab,ti,kw OR 'mental disab*':ab,ti,kw OR 'mentally disab*':ab,ti,kw OR 'mental deficien*':ab,ti,kw OR 'mentally deficien*':ab,ti,kw OR 'intellectual deficien*':ab,ti,kw OR 'mental handicap*':ab,ti,kw OR 'mentally handicap*':ab,ti,kw OR 'Intellectual Development Disorder*':ab,ti,kw OR 'intellectual impair*':ab,ti,kw OR 'intellectually impair*':ab,ti,kw OR 'intellectual dysfunct*':ab,ti,kw OR 'mental impair*':ab,ti,kw OR 'mentally impair*':ab,ti,kw OR 'mental dysfunct*':ab,ti,kw OR 'cognitive impair*':ab,ti,kw OR 'cognitive disab*':ab,ti,kw OR 'cognitive deficien*':ab,ti,kw OR 'cognitive defect*':ab,ti,kw OR 'cognition impair*':ab,ti,kw OR 'cognition deficien*':ab,ti,kw OR 'Down Syndrome':ab,ti,kw OR 'Downs Syndrome':ab,ti,kw OR 'Down s Syndrome':ab,ti,kw** | 824,889 |
| #1 | **'desensitization (psychology)'/de OR 'eye movement desensitization and reprocessing'/exp OR 'Eye Movement Desensit*':ab,ti,kw OR EMDR:ab,ti,kw** | 1,602 |

**APA PsycInfo (Ebsco) Session Results (01 May 2023)**

| Search | Query | Items found |
| --- | --- | --- |
| #3 | **#1 AND #2** | 34 |
| #2 | **DE "Cognitive Impairment" OR DE "Intellectual Development Disorder" OR DE "Down's Syndrome" OR TI ("cognitive dysfunct*" OR "intellectual disab*" OR "intellectually disab*" OR "mental disab*" OR "mentally disab*" OR "mental deficien*" OR "mentally deficien*" OR "intellectual deficien*" OR "mental handicap*" OR "mentally handicap*" OR "Intellectual Development Disorder*" OR "intellectual impair*" OR "intellectually impair*" OR "intellectual dysfunct*" OR "mental impair*" OR "mentally impair*" OR "mental dysfunct*" OR "cognitive impair*" OR "cognitive disab*" OR "cognitive deficien*" OR "cognitive defect*" OR "cognition impair*" OR "cognition deficien*" OR "Down Syndrome" OR "Downs Syndrome" OR "Down s Syndrome") OR AB ("cognitive dysfunct*" OR "intellectual disab*" OR "intellectually disab*" OR "mental disab*" OR "mentally disab*" OR "mental deficien*" OR "mentally deficien*" OR "intellectual deficien*" OR "mental handicap*" OR "mentally handicap*" OR "Intellectual Development Disorder*" OR "intellectual impair*" OR "intellectually impair*" OR "intellectual dysfunct*" OR "mental impair*" OR "mentally impair*" OR "mental dysfunct*" OR "cognitive impair*" OR "cognitive disab*" OR "cognitive deficien*" OR "cognitive defect*" OR "cognition impair*" OR "cognition deficien*" OR "Down Syndrome" OR "Downs Syndrome" OR "Down s Syndrome") OR KW ("cognitive dysfunct*" OR "intellectual disab*" OR "intellectually disab*" OR "mental disab*" OR "mentally disab*" OR "mental deficien*" OR "mentally deficien*" OR "intellectual deficien*" OR "mental handicap*" OR "mentally handicap*" OR "Intellectual Development Disorder*" OR "intellectual impair*" OR "intellectually impair*" OR "intellectual dysfunct*" OR "mental impair*" OR "mentally impair*" OR "mental dysfunct*" OR "cognitive impair*" OR "cognitive disab*" OR "cognitive deficien*" OR "cognitive defect*" OR "cognition impair*" OR "cognition deficien*" OR "Down Syndrome" OR "Downs Syndrome" OR "Down s Syndrome")** | 132,244 |
| #1 | **DE "Eye Movement Desensitization Therapy" OR TI ("Eye Movement Desensit*" OR EMDR) OR AB ("Eye Movement Desensit*" OR EMDR) OR KW ("Eye Movement Desensit*" OR EMDR)** | 2,472 |

**Web of Science (Core Collection) Session Results (01 May 2023)**

| Search | Query | Items found |
| --- | --- | --- |
| #3 | **#1 AND #2** | 45 |
| #2 | **TS=("cognitive dysfunct*" OR "intellectual disab*" OR "intellectually disab*" OR "mental disab*" OR "mentally disab*" OR "mental deficien*" OR "mentally deficien*" OR "intellectual deficien*" OR "mental handicap*" OR "mentally handicap*" OR "Intellectual Development Disorder*" OR "intellectual impair*" OR "intellectually impair*" OR "intellectual dysfunct*" OR "mental impair*" OR "mentally impair*" OR "mental dysfunct*" OR "cognitive impair*" OR "cognitive disab*" OR "cognitive deficien*" OR "cognitive defect*" OR "cognition impair*" OR "cognition deficien*" OR "Down Syndrome" OR "Downs Syndrome" OR "Down s Syndrome")** | 230,962 |
| #1 | **TS=("Eye Movement Desensit*" OR EMDR)** | 2,352 |
